# ﻿Discovery of a new *Isonychia* species with distinctive characters from southwestern China, and preliminary exploration of its phylogenetic status (Ephemeroptera, Isonychiidae)

**DOI:** 10.3897/zookeys.1218.137110

**Published:** 2024-11-19

**Authors:** Pengxu Mu, Xiaolei Huang

**Affiliations:** 1 State Key Laboratory of Ecological Pest Control for Fujian and Taiwan Crops, College of Plant Protection, Fujian Agriculture and Forestry University, Fuzhou 350002, China Fujian Agriculture and Forestry University Fuzhou China

**Keywords:** *16S*, *COI*, mayfly, molecular phylogeny, taxonomy

## Abstract

The genus *Isonychia* Eaton, 1871 is widely distributed across the Holarctic and Oriental regions. However, no representatives of this genus have been reported from southwestern China, a region known for its high biodiversity. Here, we described and illustrated *Isonychialatias***sp. nov.**, a new species recently collected from Guizhou Province, southwestern China, across all developmental stages. The imagos of this new species exhibit some uncommon characters within *Isonychia*, such as brown mid- and hindlegs, and pale stripes on the thorax. To explore the phylogenetic status of this new species within *Isonychia*, a multigene phylogenetic analysis was conducted.

## ﻿Introduction

Isonychiidae is a monogeneric mayfly family represented by a single extant genus, *Isonychia* Eaton, 1871, which includes 16 species distributed in the Nearctic region and 21 species distributed in the Palaearctic and Oriental regions (Tiunova et al. 2004; Muthukatturaja et al. 2021; Huang et al. 2024; Qiang et al. 2024).

Kondratieff and Voshell (1983) conducted a comprehensive classification of the *Isonychia* species in North America. These authors established a new subgenus, *Prionoides* Kondratieff & Voshell, 1983 and divided the *Isonychia* s.s. into four species groups, including non-Nearctic species. McCafferty (1989) erected a new subgenus, *Borisonychia* McCafferty, 1989, for *I.diversa* Traver, 1934, the only representative of the *diversa* group sensu Kondratieff and Voshell (1983). Unfortunately, *I.diversa* has been declared extinct (McCafferty 2001), and no new members of this subgenus have been identified since then.

Before this study, a total of nine *Isonychia* species had been recognized from China (Qiang and Zhou 2023; Huang et al. 2024; Qiang et al. 2024), including three species from northeastern China (*I.sexpetala*Tiunova et al., 2004, *I.ussurica* Bajkova, 1970 and *I.vshivkovae*Tiunova et al., 2004), four species from eastern China (*I.formosana* (Ulmer, 1912), *I.guixiensis*Wu et al., 1992, *I.kiangsinensis* Hsu, 1936 and *I.taishunensis*Huang et al., 2024), and two species from southern China (*I.ignota* (Walker, 1853) and *I.fuscimarginata*Qiang et al., 2024). Thus, the fauna of western China for this genus remains unknown.

Recently, we conducted a preliminary investigation of the mayfly fauna in Guizhou Province, southwestern China, and discovered an undescribed species of *Isonychia*. This species exhibits several uncommon characters within the genus. To determine its status within *Isonychia*, we performed a multigene phylogenetic analysis alongside adequate morphological studies.

## ﻿Materials and methods

The adults and larvae of the new species were collected from the same site in Zunyi City, Guizhou Province, and were associated by DNA barcoding based on the mitochondrial cytochrome c oxidase subunit I (COI) gene. All imagos were reared from subimagos caught using a light trap, and the larval exuviae were found on rocks in a stream. All specimens used in this study are preserved in 95% ethanol and are deposited in the State Key Laboratory of Ecological Pest Control for Fujian and Taiwan Crops, College of Plant Protection, Fujian Agriculture and Forestry University (**FAFU**).

The specimens were examined and photographed using a computer-connected Nikon SMZ18 stereomicroscope. The photos were processed with Adobe Photoshop CC 2019. The SEM samples were dehydrated in 100% ethanol for 15 min and then coated with gold film in a vacuum.

To explore the status of our new species within *Isonychia*, we conducted a multigene phylogenetic analysis using the mitochondrial genes *COI* and 16S ribosomal RNA (16S). Total DNA was extracted from legs of larvae or adults using Trelief Hi-Pure Animal Genomic DNA Kit (Tsingke, Beijing, China). The mitochondrial genes *COI* and *16S* were PCR-amplified with the primers specified in Folmer et al. (1994) and Ogden and Whiting (2005), respectively. Optimized PCR conditions were as follows: 30 s of initial denaturation at 98 °C, a total of 35 cycles with denaturation at 98 °C for 10 s, annealing at 52 °C for 30 s and an extension at 72 °C for 30 s, and 2 min of final extension at 72 °C. The products of PCR were bidirectionally sequenced at Tsingke Biotechnology (Beijing, China). All sequences obtained in this study were assembled using BioEdit (Hall 1999) and deposited in GenBank. The accession numbers, along with the GPS coordinates of sample locations, are provided in Table 1. The nomenclature of gene sequences follows Chakrabarty et al. (2013). In addition to the sequences of our new species, we included sequences of five *Isonychia* species and two non-*Isonychia* species from GenBank for the phylogenetic analysis; the details of these sequences are shown in Table 2.

**Table 1.** Sequenced specimens of *Isonychialatias* sp. nov. (“-” indicates the same content as above).

Specimen voucher	Locality	Coordinates	Date	Stage	GenBank #	GenSeq nomenclature
GZZY01BaN011A1	Xishui, Guizhou	28.497144N, 106.410003E	21.V.2024	subimago	PP922980	genseq-2 COI
-	-	-	-	-	PQ289235	genseq-2 16S
GZZY01BaN011L2	-	-	-	larva	PP922981	genseq-2 COI

**Table 2.** Sequences of *Isonychia* spp. obtained from GenBank.

Species	Gene	GenBank #	Reference	Notes
I. (Prionoides) shima	*COI*	LC106878	Saito and Tojo 2016	—
I. (Prionoides) shima	*16S*	LC106655	Saito and Tojo 2016	—
I.(s.s.)japonica	*COI*	LC106699	Saito and Tojo 2016	As *I.valida*
I.(s.s.)japonica	*16S*	LC106476	Saito and Tojo 2016	As *I.valida*
I.(s.s.)ignota	*COI*	LC114396	Saito et al. 2016	—
I.(s.s.)ignota	*16S*	LC114375	Saito et al. 2016	—
I.(s.s.)ussurica	*COI*	LC114401	Saito et al. 2016	—
I.(s.s.)ussurica	*16S*	LC114379	Saito et al. 2016	—
I.(s.s.)kiangsinensis	*COI*/*16S*	MH119135	Ye et al. 2018	Derived from mitogenome
*Chromarcysmagnifica*	*COI*	MG516472	Massariol et al. 2019	—
*Chromarcysmagnifica*	*16S*	MG516460	Massariol et al. 2019	—
*Paegniodescupulatus*	*COI*/*16S*	MW381300	Li et al. 2021	Derived from mitogenome

The phylogenetic analysis was conducted using the integrated platform PhyloSuite v. 1.2.3 (Xiang et al. 2023). Multiple sequence alignments were performed using MAFFT (Katoh and Standley 2013) in “Normal” alignment mode. Further trimming of the *COI* and *16S* alignments was carried out using trimAl (Capella-Gutierrez et al. 2009) with default parameters before concatenation. The concatenated alignments were then imported into ModelFinder (Kalyaanamoorthy et al. 2017) to select the best-fit models for phylogenetic estimates using the “Edge-linked” partition mode. According to the Bayesian Information Criterion, the best fit models were TIM2+F+G4 for *COI* and TPM2u+F+G4 for *16S*. Maximum-likelihood (ML) phylogenetic tree reconstruction was conducted using IQ-TREE (Minh et al. 2020), with branch support analysis performed in “Ultrafast” mode with 5,000 bootstraps; other parameters were set to default. The phylogenetic tree was visualized using iTOL v. 6 (Letunic and Bork 2024).

Terminology for egg structure followed Koss and Edmunds (1974); the term “microlepides” was used according to Kluge and Novikova (2011); other terms were used according to Kluge (2004).

## ﻿Results

### ﻿ Isonychia latias sp. nov.

Taxon classificationAnimaliaEphemeropteraIsonychiidae

63D6E8E3-7D51-5808-829E-22D61CB7A3E6

https://zoobank.org/BDD9DBA9-82B4-4D68-BCF8-42F53CEC10A2

Figs 1 , 2 , 3 , 4 , 5 , 6 , 7 , 8 , 9 Chinese name: 比翼等蜉

#### Type material.

***Holotype*: male imago, China** • **Guizhou Province**, Zunyi City, Xishui County, China Dan Xia Valley, Sanchahe River [贵州省遵义市习水县中国丹霞谷三岔河] (28.497144N, 106.410003E, alt. 880 m), 21.V.2024, leg. Pengxu Mu; in ethanol; FAFU. ***Paratypes*** • 8 male imagos, 8 female imagos, 12 male subimagos, 15 female subimagos, 12 larvae, 22 larval exuviae, same information as holotype; in ethanol; FAFU.

#### Description.

**Male imago.** Forewing length 18.5–20.0 mm.

**Figure 1.**
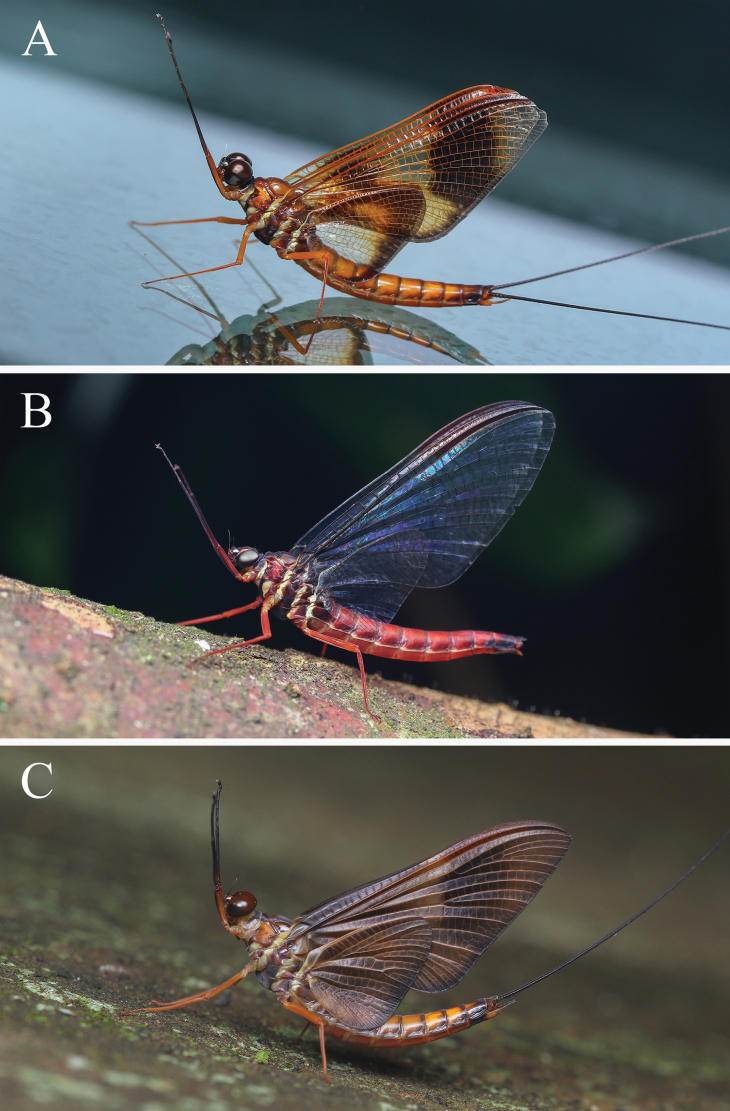
Adults of *Isonychialatias* sp. nov. **A** male imago **B** female imago **C** male subimago. (Photographed by Qianle Lu).

**Figure 2.**
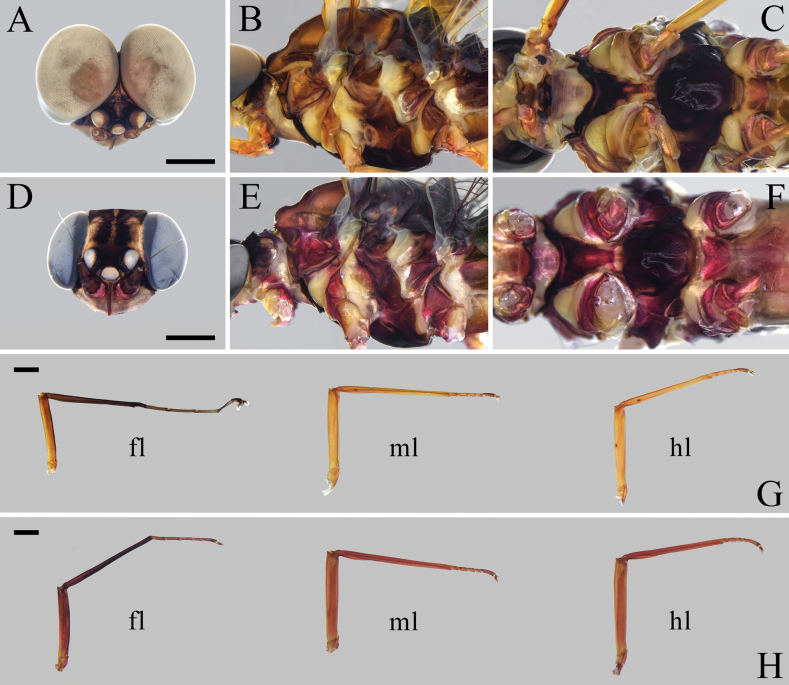
Imaginal structures of *Isonychialatias* sp. nov. **A–C** male imago **A** head **B** thorax, lateral view **C** thorax, ventral view **D–F** female imago **D** head **E** thorax, lateral view **F** thorax, ventral view **G, H** legs (fl: foreleg; ml: midleg; hl: hindleg) **G** male imago **H** female imago. Scale bars: 1.0 mm (**A, D, G, H**).

**Figure 3.**
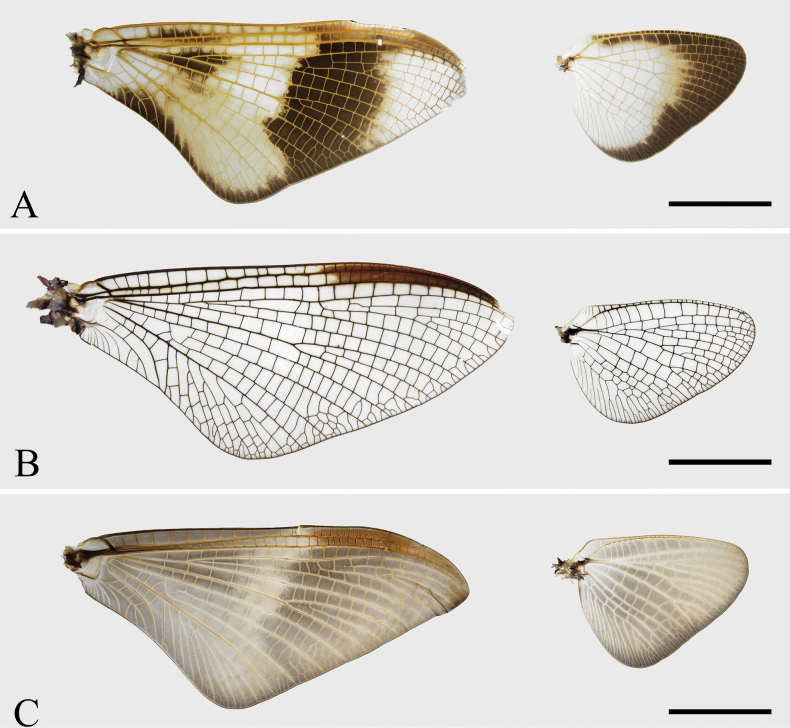
Wings of *Isonychialatias* sp. nov. **A** male imago **B** female imago **C** male subimago. Scale bars: 5.0 mm.

**Figure 4.**
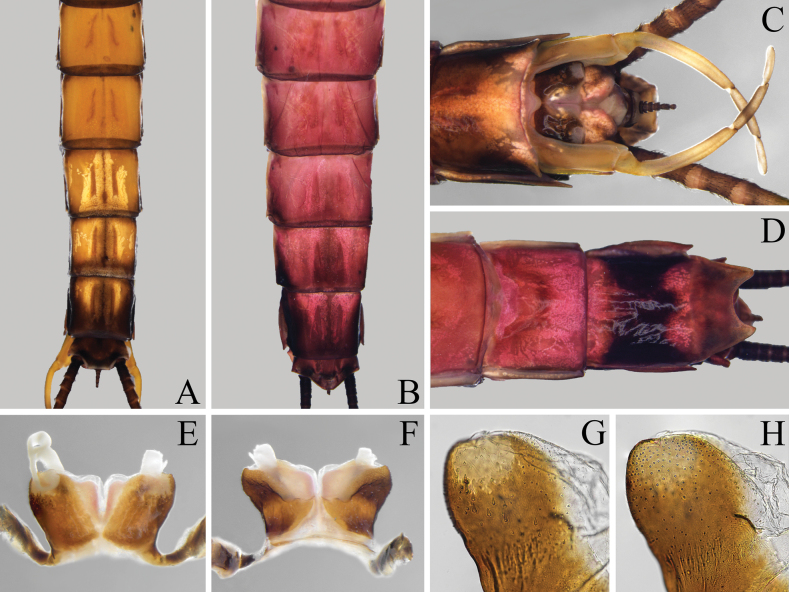
Imaginal structures of *Isonychialatias* sp. nov. **A, B** abdominal segments V–X, dorsal view **A** male **B** female **C, D** genital segments, ventral view **C** male **D** female **E–H** penis **E** ventral view **F** dorsal view **G** penis lobe enlarged, ventral view **H** penis lobe enlarged, dorsal view.

***Coloration*.** General body color brown to reddish brown (Fig. 1A). Head capsule dark brown except anterior part pale; compound eyes gray (Fig. 2A). Pronotum dark brown except posterolateral parts pale; mesoscutum brown, and mesoscutellum and metanotum dark brown; pleura of pterothorax with three yellowish stripes formed by pale conjunctivas; basisternum and furcasternum of mesothorax dark reddish brown, and basisternum of metathorax pale reddish (Fig. 2B, C). Forefemur brown, slightly shaded with dark brown apically; foretibia and foretarsus dark brown; mid- and hindlegs brown (Fig. 2G). Fore- and hindwings with distinct, dark brown coloration and pale yellowish shadings as in Fig. 3A; all veins of both wings pale brown. Abdominal terga I and X dark brown; terga II–VII pale brown, except lateral margins slightly shaded with dark brown; terga VIII–IX with anteromedian part pale brown and posterolateral part dark brown, and dark brown area of tergum IX larger than that of tergum VIII; terga II–IX with pair of dark, submedian, longitudinal, oblique stripes (Fig. 4A). Abdominal sterna reddish brown except sternum IX dark brown; sterna II–IX with pair of dark submedian longitudinal oblique stripes, and sterna II–VI with four dark dots situated in transverse line behind these stripes. Styliger and gonostyli pale brown, and penis dark brown (Fig. 4C). Cerci dark brown along their entire length.

***Legs*** (Fig. 2G). Foreleg: length ratio of femur (2.7 mm):tibia:tarsus 1.0:1.5:1.6, length ratio of tarsomeres from basal to apical 1.0:1.3:1.0:0.9:0.4. Midleg: length ratio of femur (3.2 mm):tibia:tarsus 1.0:1.4:0.6, tarsomeres arranged in decreasing order as 5, 2, 1, 3, 4. Hindleg: length ratio of femur (3.2 mm):tibia:tarsus 1.0:1.2:0.5, tarsomeres arranged in decreasing order as 5, 2, 1, 3, 4. Foreleg with both claws similar, blunt, and provided with a soft plate; mid- and hindlegs with both claws similar and pointed.

***Wings*** (Fig. 3A). Forewing: number of crossveins relatively large, and pterostigmatic area with about 30 crossveins between C and Sc; MP forked asymmetrically, MP_2_ strongly curved in proximal part; cubital field with two or three unforked and four forked veins gone from CuA to basitornal and tornoapical margins. Hindwing: length ratio of maximum length:width 1.4:1.0; costal projection round and with 4–5 crossveins; tornoapical margin slightly concave; RS forked about 1/2 of distance from base of vein to margin; MA forked slightly more apically than RS; MP forked about 3/4 of distance from base of vein to margin.

***Genitals*** (Fig. 4C, E–H). Gonostyli pedestals relatively long with blunt ventral-apical-median angles. Gonostylus with length of segment II ca 2.0 of segment III length, and length of segment III ca 1.4 of segment IV length. Penis reaching to middle part of gonostyli pedestals; penis lobes deeply separated with apices strongly divergent, and stout spines only present on subapical area of ventral surface. Processes between styliger and penis absent.

**Female imago.** Forewing length 22.7–23.5 mm. Similar to male imago except the following:

***Coloration*.** General body color reddish (Fig. 1B). Compound eyes blue-gray (Fig. 2D). Thoracic pattern similar to male imago but reddish in general (Fig. 2E, F). Forefemur reddish, slightly shaded with dark reddish apically; foretibia dark reddish; foretarsus, mid- and hindlegs reddish (Fig. 2H). Forewing with dark brown band occupying pterostigmatic area; hindwing colorless; all veins of both wings dark brown (Fig. 3B). Abdominal tergum I dark reddish; terga II–VII reddish; terga VIII–IX with anteromedian part reddish and posterolateral part black, and black area of tergum IX larger than that of tergum VIII (Fig. 4B). Abdominal sterna reddish brown, except sternum IX with large, transverse, dark brown band (Fig. 4D).

***Legs*** (Fig. 2H). Foreleg: length ratio of femur (2.8 mm):tibia:tarsus 1.0:1.5:0.9, length ratio of tarsomeres from basal to apical 1.0:1.1:0.9:0.7:1.1. Midleg: length ratio of femur (3.5 mm):tibia:tarsus 1.0:1.3:0.5. Hindleg: length ratio of femur (3.8 mm):tibia:tarsus 1.0:1.0:0.4. Both claws of all legs similar and pointed.

***Wings*** (Fig. 3B). Hindwing: length ratio of maximum length:width 1.6:1.0; RS forked about 2/5 of distance from base of vein to margin; MA forked about 1/2 of distance from base of vein to margin; MP forked about 7/10 of distance from base of vein to margin.

***Genitalia*** (Fig. 4D). Subgenital plate slightly elongated with rounded posterior margin. Subanal plate with deep posteromedian emargination.

**Male subimago.** Similar to male imago except the following: Mesonotum with brown lateral pigmented area occupying submedioscutum and sublateroscutum back to posterior scutal protuberance (PSp); medioscutum and PSp pale. Foreleg: length ratio of femur (2.6 mm):tibia:tarsus 1.0:1.4:1.2, length ratio of tarsomeres from basal to apical 1.0:1.0:0.8:0.7:0.7. Tarsomeres of all legs covered with “U”-shape, blunt microlepides. Both claws of all legs similar and pointed. Wings brown to dark brown in general, coloration as in Fig. 3C.

**Female subimago.** Similar to male subimago except the following: Foreleg: length ratio of femur (2.9 mm):tibia:tarsus 1.0:1.4:0.9, length ratio of tarsomeres from basal to apical 1.0:1.1:0.9:0.7:1.1. Subgenital plate not elongated.

**Larva.** Body length: male 19.3–20.6 mm; female 23.7–26.1 mm.

**Figure 5.**
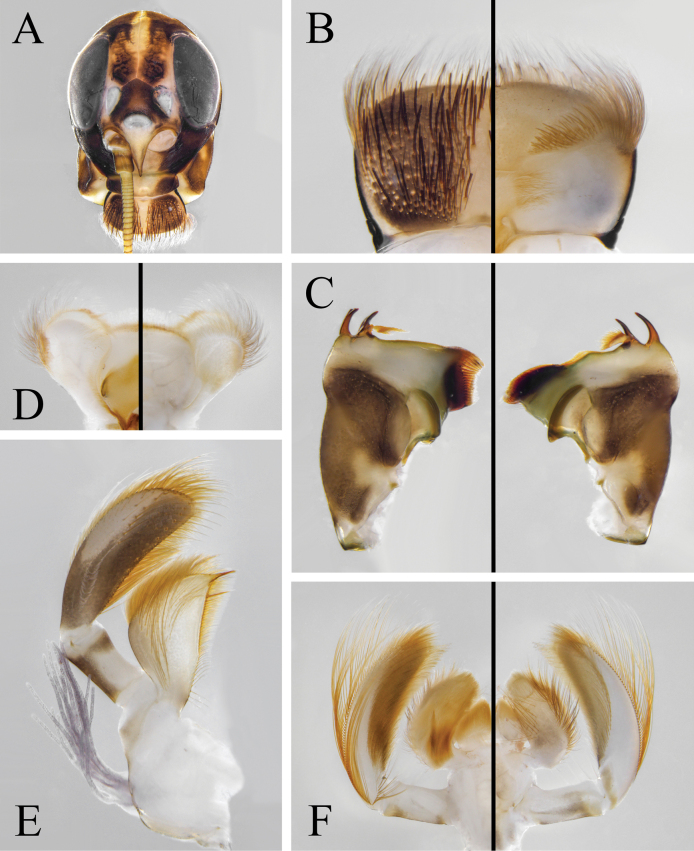
Larval structures of *Isonychialatias* sp. nov. **A** head, front view **B** labrum (left: dorsal view; right: ventral view) **C** mandible, dorsal view (left: left mandible right: right mandible) **D** hypopharynx (left: dorsal view right: ventral view) **E** maxilla, dorsal view **F** labium (left: dorsal view; right: ventral view).

**Figure 6.**
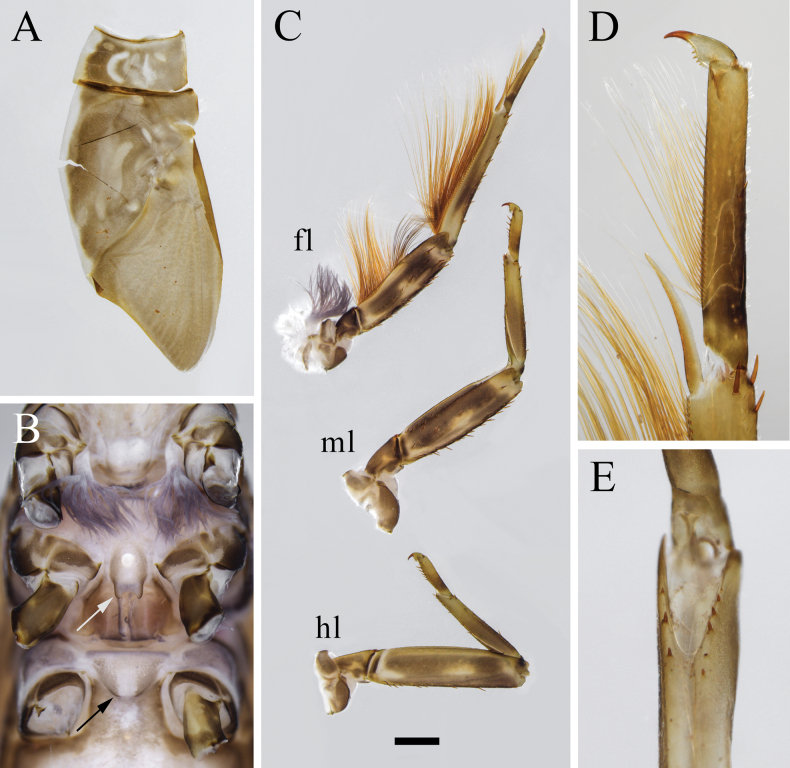
Larval structures of *Isonychialatias* sp. nov. **A** right half of pronotum and mesonotum **B** thorax, ventral view (white arrow shows projection on basisternum of mesothorax black arrow shows projection on basisternum of metathorax) **C** legs (fl: foreleg ml: midleg hl: hindleg) **D** apical part of foreleg **E** ventral cleft of hindfemur. Scale bar: 1.0 mm (**C**).

**Figure 7.**
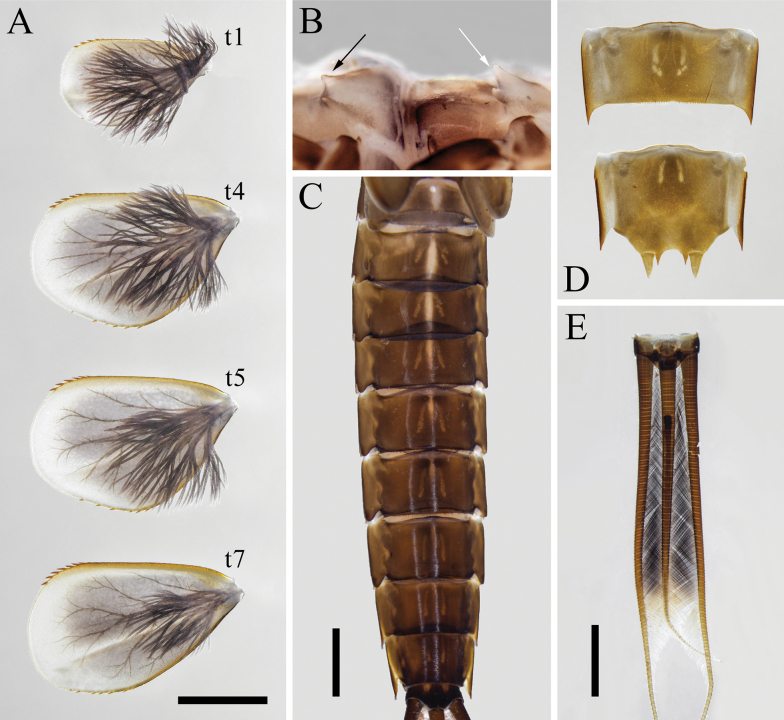
Larval structures of *Isonychialatias* sp. nov. **A** tergalii (t1: tergalius I; t4: tergalius IV; t5: tergalius V; t7: tergalius VII) **B** thoracic sterna, lateral view (white arrow shows projection on basisternum of mesothorax black arrow shows projection on basisternum of metathorax) **C** abdominal terga I–X **D** abdominal sterna VIII–IX **E** caudalii. Scale bars: 1.0 cm (**A**); 2.0 mm (**C, E**).

***Coloration*.** General body color dark brown. Head capsule dark brown, except frontal carina, and median parts of clypeus and vertex yellowish; scape and pedicel brown, flagella pale; dorsum of labrum dark brown with pale longitudinal line medially (Fig. 5A). Thoracic nota dark brown but with pale median longitudinal stripe and irregular pale markings (Fig. 6A). Forefemur with two transverse dark bands, foretibia with 1 transverse dark band medially, and foretarsus with 1 transverse dark band in proximal part; mid- and hindleg with similar coloration except femur with two transverse dark bands connected by dark stripe, and dark band on tibia more basally (Fig. 6C). Abdominal terga dark brown in general; terga I–VII with pale median longitudinal stripes (shorter and less pronounced posteriorly); terga II–IX with pair of pale submedian longitudinal stripes; tergum X with pair of light spots close to anterior margin (Fig. 7C). Abdominal sterna brown in general; sterna II–IX with pair of pale submedian longitudinal stripes, and sterna II–VIII with four dots situated in transverse line behind these stripes. Tergalii without distinct dots or markings (Fig. 7A). Caudalii brown basally and gradually paler towards apices without dark band medially.

***Mouthparts*** (Fig. 5B–F). Typical of *Isonychia*, setal pattern consistent with other congeners in general. Labrum subquadrate, widest part about twice as long. Superlingua round, width ca 0.6 of lingua width. Distal dentiseta of maxilla strongly diminished, needle-like, distinctly shorter and slenderer than proximal dentiseta. Length of segment I of maxillary palp ca 0.4 of segment II length. Length of segment I of labial palp ca 0.5 of segment II length. Length of paraglossa ca 0.5 of glossa length.

***Legs*** (Fig. 6C–E). Setal pattern typical of *Isonychia*. Ventral cleft of hind femur with 5–8 spines. All claws with 7–9 blunt denticles. Gill on joining of forecoxa with thorax well developed, tuft-like.

***Thoracic sterna*** (Figs 6B, 7B). Bifurcate projection on basisternum of mesothorax well developed; paired projections on basisternum of metathorax relatively weakly expressed.

***Tergalii*** (Fig. 7A). Lamella of tergalius I distinctly smaller than other lamellae of tergalii, apical part of costal rib with 4–5 spine-like denticles; lamellae of tergalii II–VII gradually larger posteriorly, each lamella usually with 5–7 spine-like denticles on apical part of costal rib and on apical part of posterior branch of anal rib (rarely beyond this range, and in tergalii III–VII mostly with 6 denticles), and no denticles present on apical margin between these two areas. Ventral fibrillose lobe well developed in all tergalii.

***Abdominal terga and sterna*** (Fig. 7C, D). Terga II–X with acute denticles along posterior margins. Sterna VI–VIII with acute denticles along posterior margins, and subanal plate with smaller and denser denticles on median part of posterior margin. Posterolateral spines well developed on segment VIII–IX.

**Figure 8.**
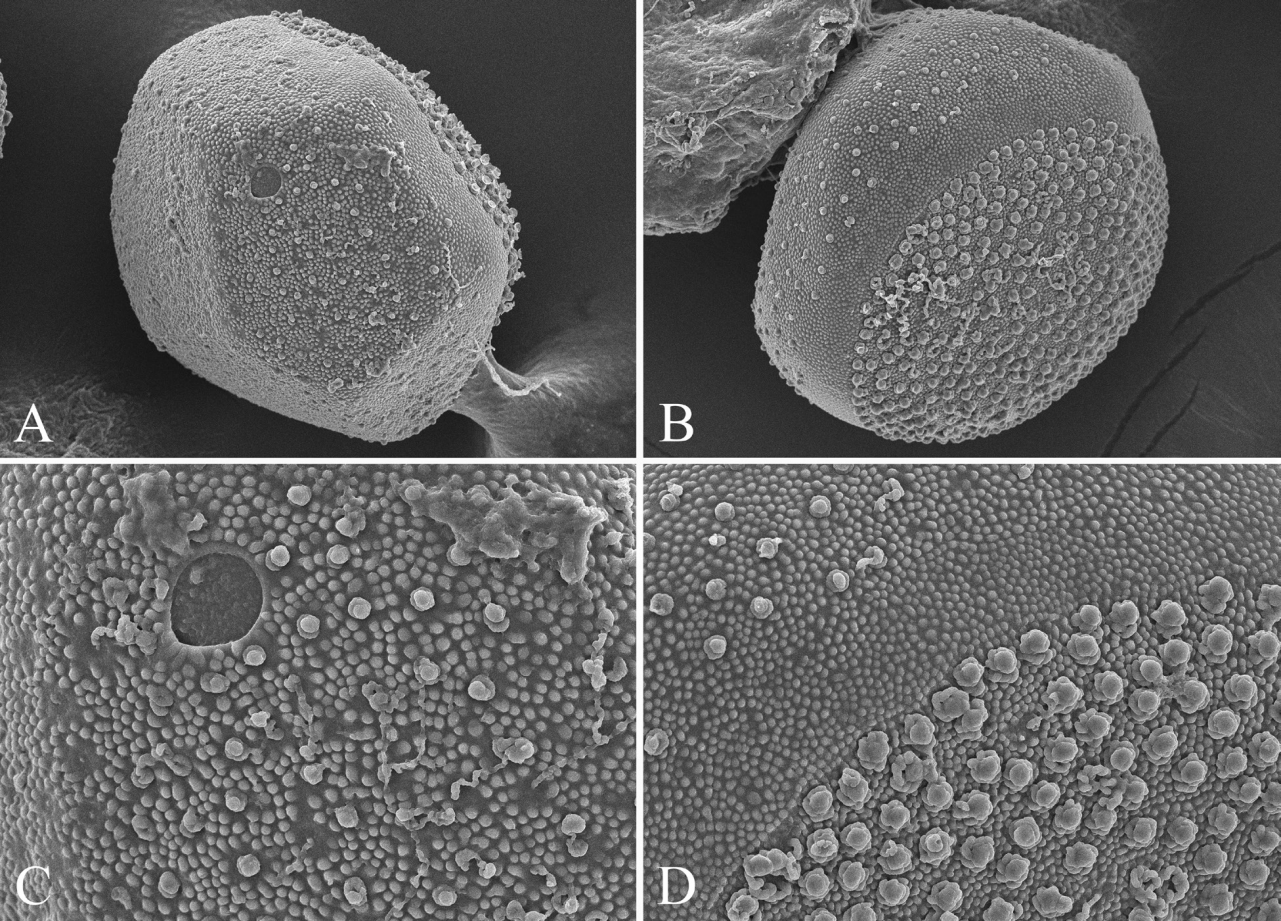
SEM photos of eggs of *Isonychialatias* sp. nov. **A, B** egg **C** micropyle enlarged **D** KCTs enlarged.

**Egg** (Fig. 8A–D). Spherical; chorion densely covered with small tubercles, and without reticulation; KCTs dense in one hemisphere, and other area sparsely scattered with smaller KCTs.

#### Diagnosis.

*Isonychialatias* sp. nov. can be readily distinguished from its congeners by the following combination of characters: For male imago: A) wings with distinct, dark brown coloration: on forewing occupying a large area of apical half as a transverse band, a small area around bifurcation point of Rs, and edges along basitornal margin and tornus, and on hindwing occupying almost whole apical part; B) mid- and hindlegs brown, nearly consistent with forefemur; C) pleura of pterothorax with three yellowish stripes formed by pale conjunctivas. For larvae: A) lamellae of all tergalii without spines on apical margin, and without distinct dots or markings; B) abdominal terga I–VII with pale median longitudinal stripes, terga II–IX with pair of pale submedian longitudinal stripes, and tergum X with pair of light spots close to anterior margin; C) caudalii brown basally and gradually paler towards apices without dark band medially. The larvae of *I.latias* sp. nov. resemble those of *I.fuscimarginata*Qiang et al., 2024, based on the similar coloration of abdominal terga. However, they can be differentiated by the following characters: A) each lamella of tergalii of *I.fuscimarginata* with a large middle dark purple dot basally, while those of *I.latias* sp. nov. without dots; B) ventral fibrillose lobes of tergalii of *I.fuscimarginata* with fewer filaments than those of *I.latias* sp. nov.; C) *I.fuscimarginata* with submedian dark band on all caudalii and apical dark band on cerci, while all caudalii of *I.latias* sp. nov. without dark band along their entire length.

#### Distribution.

China: Guizhou Province (Zunyi City, Sanchahe River).

#### Etymology.

The new species is named after Latias, an alate Pokémon with red and white appearance. The specific epithet *latias* is treated as a noun in apposition to the generic name.

#### Biology.

**Figure 9.**
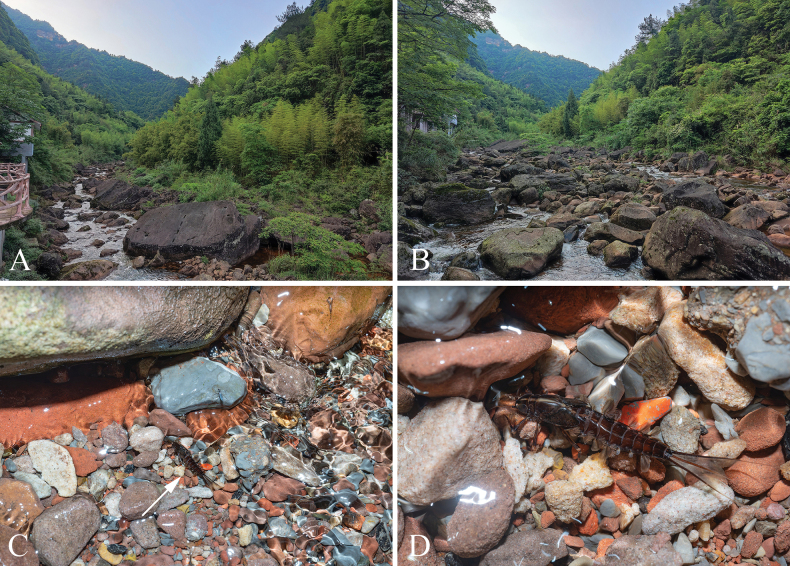
Habitat of *Isonychialatias* sp. nov. **A, B** Sanchahe River, Dan Xia Valley, China **C, D** mature larva of *Isonychialatias* sp. nov. about to emerge in natural environment.

The larvae of *Isonychialatias* sp. nov. have so far been found only in the Sanchahe River in Guizhou Province. The collection site is located near a Danxia landform, characterized by a large amount of dark red rocks in the river (Fig. 9A, B). The larvae were primarily collected in rapid sections, and one mature larva about to emerge was found in a gravelly shallow area (Fig. 9C, D). The exuviae of the larvae were mainly found on stones 10–20 cm above the water surface, with the highest reaching more than 50 cm. The subimagos of both sexes moulted into imagos spending three nights after they were caught.

##### ﻿Genetics

**Figure 10.**
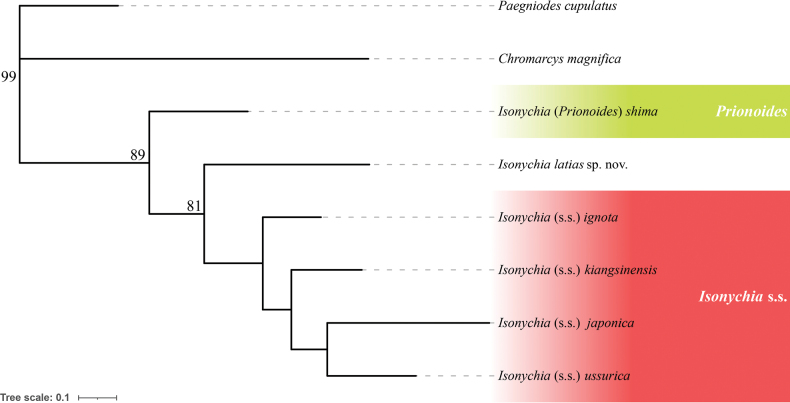
ML tree for *Isonychia* spp based on concatenated sequences of two genes (*COI* and *16S*) showing *Prionoides* (green) and *Isonychia* s.s. (red). ML bootstrap values above 70 are indicated next to the nodes.

We performed a multi‐gene phylogenetic analysis using the mitochondrial genes *COI* and *16S*, including our new species, four *Isonychia* s.s. species, and one *Prionoides* species (Table 2). *Chromarcysmagnifica* Navás, 1932 and *Paegniodescupulatus* (Eaton, 1871) were used as outgroups. The topology of the ML tree shows that Isonychia (Prionoides) shima (Matsumura, 1931) is the first species to branch out, forming a sister group with the remaining species. Within the remaining species, our new species is the first to split off, forming a sister group with the other four *Isonychia* s.s. species, which form a monophyletic group (Fig. 10).

## ﻿Discussion

Tiunova et al. (2004) revised the *Isonychia* species of the Eastern Palaearctic region, reviewing the concept of *Isonychia* along with its two subgenera, *Isonychia* s.s. and *Prionoides*. In general, *I.latias* sp. nov. apparently morphologically belongs to *Isonychia* s.s., based on its spherical eggs, the strongly reduced styliger in male adults and the tuft-like forecoxal gills in larvae. This result is consistent with our molecular phylogenetic analysis, which shows that *I.latias* sp. nov. is grouped with four other *Isonychia* s.s. species, together forming a sister clade with I. (P.) shima.

However, it is worth mentioning that some of the reviewed characters given by Tiunova et al. (2004) do not apply to our new species. First, Tiunova et al. argued that forewing coloration could be present in male imagos of some *Isonychia* species but always absent in their female imagos. This is not the case in *I.latias* sp. nov., where the female imago has dark brown coloration occupying the pterostigmatic area. Such exceptions are also found in *I.formosana* (Ulmer, 1912) and *I.fuscimarginata*Qiang et al., 2024 (the female imago of the former has larger colored area than male on both wings; the female imago of the latter has similar wing coloration with male). Second, Tiunova et al. indicated that the first tarsomere is equal to or slightly longer than the second one in imagos of both sexes. In contrast, the first tarsomere of the male imago of *I.latias* sp. nov. is shorter than second one (length ratio of tarsomere I:II 1.0:1.3 in the holotype and 1.0:1.4 in one paratype).

Besides these two characters, the coloration of the legs and pleura of our new species is also uncommon in *Isonychia*. In most *Isonychia* species (at least in all well-studied *Isonychia* s.s. species), the mid- and hindlegs of imagos are distinctly paler than forelegs, and the coloration of pleura is relatively uniform. However, *I.latias* sp. nov. has brown to reddish brown mid- and hindlegs consistent with the forefemur, and its pleura of the pterothorax is more colorful. Notably, similar coloration was found in some *Prionoides* species, such as I. (P.) shima from Japan (Saito et al. 2020: fig. 1B). Correspondingly, the position of *I.latias* sp. nov. in our phylogenetic tree indicates its potential ancestral status within the *Isonychia* s.s clade. However, our molecular phylogenetic analysis did not include enough species to draw a definitive conclusion on the exact position of our new species within *Isonychia*. More research, especially of the Oriental fauna, is needed to fully understand the evolution of *Isonychia* and the systematic position of *I.latias* sp. nov. within the genus.

## Supplementary Material

XML Treatment for Isonychia latias

## ﻿Additional information

### Author ORCIDs

Pengxu Mu https://orcid.org/0000-0003-4906-0955

Xiaolei Huang https://orcid.org/0000-0002-6839-9922

### Data availability

All of the data that support the findings of this study are available in the main text.
